# Spatially restricted coral bleaching as an ecological manifestation of within-colony heterogeneity

**DOI:** 10.1038/s42003-025-08150-4

**Published:** 2025-05-13

**Authors:** Christian R. Voolstra, Marlen Schlotheuber, Emma F. Camp, Matthew R. Nitschke, Sebastian Szereday, Sonia Bejarano

**Affiliations:** 1 Department of Biology, University of Konstanz, Konstanz, Germany; 2 Climate Change Cluster, University of Technology Sydney, Broadway, NSW Australia; 3 Australian Institute of Marine Science, Townsville, Australia; 4 School of Biological Sciences, Victoria University of Wellington, Wellington, New Zealand; 5 Coralku Solutions, Non-Profit Organization for Coral Reef Research and Restoration, Kuala Lumpur, Malaysia; 6 Reef Systems Research Group, Leibniz Centre for Tropical Marine Research (ZMT), Bremen, Germany

**Keywords:** Ecology, Tropical ecology, Ecophysiology

## Abstract

Coral bleaching is a widespread stress response of reef-building corals to elevated sea temperatures, resulting in the loss of symbiotic algae and often leading to coral death and reef degradation. Although coral bleaching occurs globally, not all reefs, species, colonies, or polyps bleach equally. Understanding intra-colony bleaching heterogeneity is crucial to anticipate the extent of coral loss at 2°C warming and harness variability to inform restorative interventions. Partially bleached coral colonies are commonly documented yet rarely tracked to determine whether they reflect ecologically distinct heterogeneity (e.g., in thermal tolerance) or eventually bleach completely. Focusing on bleaching that appears restricted to certain areas within a coral colony, we examine its putative basis in the spatial variability of the holobiont. A coral’s three-dimensional structure creates mosaics of microenvironments. Adaptations to these microenvironments are underpinned by intra-colony differences in Symbiodiniaceae association, microbiome assemblage, and nutritional status, giving rise to microhabitats. Genetic mosaicism and epigenetic changes further contribue to intra-colony phenotypic heterogeneity. We pinpoint methodologies to align spatially restricted bleaching to different forms of coral surface heterogeneity, examine the common assumption that coral fragments represent entire colonies, and illuminate implications for coral biology and restoration.

## Abstract (web summary)

This perspective highlights spatially restricted coral bleaching as an underexplored ecological phenomenon that offers critical insight into within-colony heterogeneity.

## Introduction

Global warming poses the greatest existential threat to coral reefs this century^1,2^. Triggered majoritively by heat waves coupled with high irradiance and superimposed on continued warming trends, bleaching is the clearest manifestation of the vulnerability of corals to climate change^3,4^. Bleaching occurs as the thermal tolerance limits of reef-building corals are surpassed, breaking down their symbiosis with photosynthetic Symbiodiniaceae, which supply energy and color^5,6^. Since 1998, extremely strong marine heat waves, for instance during El Niño, have triggered global-scale coral bleaching and mortality, profoundly transforming coral reefs globally^7–9^. Arguably one of the most extensively studied phenomena on reefs, coral bleaching has been recognized as spatially heterogeneous at multiple scales, from ocean basins to sites to reefs, as well as variable across and within species, populations, and genotypes^10–15^. Collectively, bleaching assessments advance our understanding of how local stressors interact with global thermal stress, locate bleaching hotspots and refugia against coral bleaching, point at traits or environmental conditions that affect bleaching responses, and thus inform spatial conservation planning, prioritization, or decision-making^10,16–20^.

Importantly, bleaching does vary at the finest of spatial scales: within single coral colonies. Partially bleached colonies (i.e., colonies that are pale or bleached only in certain areas) are in fact commonly observed and regularly documented as part of a typical bleaching assessment^21–24^. This raises the question of why heat stress triggers bleaching (at least initially) in some areas of a colony but not in others, provided that eventually the entire colony will bleach under ongoing thermal stress. Although partial bleaching is ascribed to a number of causes^11,25^, a systematic assessment is missing. This is particularly true with regard to the commonality of partial bleaching, whether partial bleaching is simply an intermediate step in the progression towards whole-colony bleaching (which may be distinct from whole-colony paling), or whether partially bleached corals are indicative of thermal stress tolerance heterogeneity across the colony surface.

Through this perspective, we review documented cases of partial colony bleaching within the published literature and differentiate cases of spatially restricted bleaching from the successive and even loss of coloration across the entire colony surface. We then pinpoint possible causes of spatially restricted bleaching rooted in the complexity and heterogeneity of the coral holobiont. Lastly, we highlight the putative consequences arising from a revised understanding of how coral colonies bleach in light of holobiont surface heterogeneity. We place an emphasis on identifying key implications for the conservation and restoration of coral in a warming ocean. For instance, if different regions within coral colonies differ in their tolerance to thermal stress, an assessment of these differences is pivotal to guiding coral reef restoration efforts to source nursery-reared coral fragments from those areas of a colony that are most resilient.

## Spatially restricted bleaching: an ecological manifestation of within-colony heterogeneity?

**Fig. 1:**
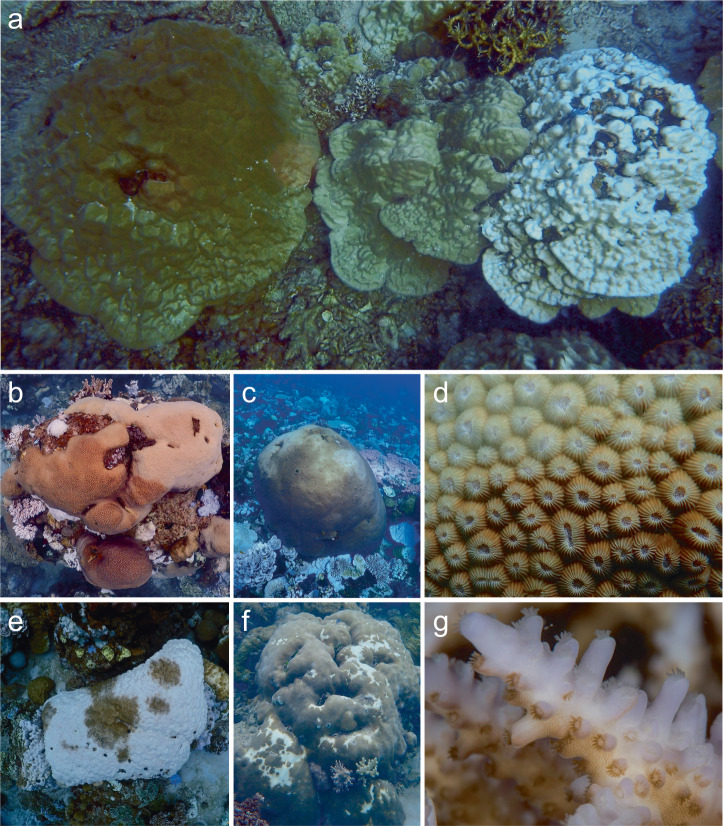
Colony bleaching heterogeneity. **a** Non-bleached colony (left), whole-colony paling (middle), and whole-colony bleaching (right). **b**–**d** Examples of spatially restricted paling where certain parts of a coral colony pale while neighboring areas remain fully colored or unaffected in the form of (**b**) partial colony paling, (**c**) spotted paling, and (**d**) paling vs. non-paling of neighboring polyps. **e**–**g** Examples of spatially restricted bleaching where certain parts of a colony are fully bleached (white) while neighboring areas remain fully colored or unaffected in the form of (**e**) partial colony bleaching, (**f**) spotted (salt-and-pepper) bleaching, and (**g**) polyp-scale bleaching where some polyps show fully bleached tentacles with neighboring polyps remaining fully pigmented. These examples illustrate that bleaching heterogeneity exists between and within colonies and may reflect spatial differences in the response to thermal stress, which may be phenomenologically different from successive whole-colony paling and bleaching. Photo credits: **a**–**e** - Sebastian Szereday; **f** - Emma Camp; **g** - Anna Roik.

**Fig. 2:**
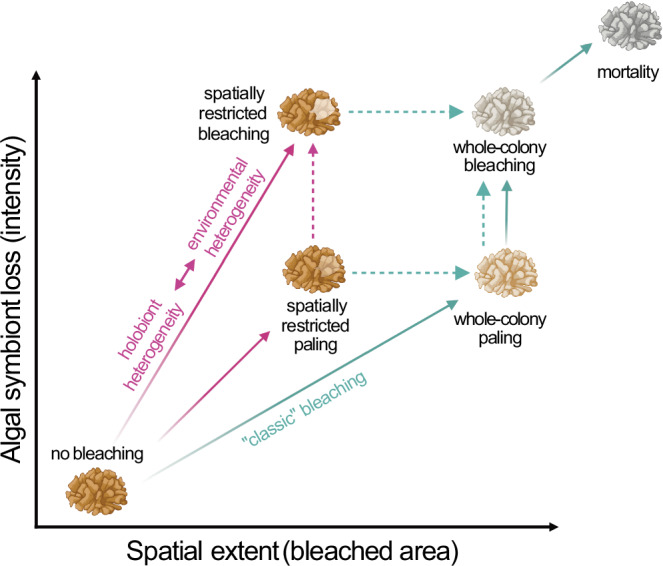
Conceptual depiction of the different types of bleaching. Bleaching can be quantified in terms of the spatial extent (i.e., bleached area, *x* axis) and the degree of algal symbiont loss (i.e., intensity, *y* axis). Based on a qualitative literature review and our own field observations (Fig. 1), we argue that different types of bleaching exist. Whole-colony paling is typically a transitory state to whole-colony bleaching triggered by continuous heat stress (green arrows). In contrast, spatially restricted paling and bleaching (purple arrows) may represent an ecologically distinct phenomenon that arises due to coral holobiont and environmental heterogeneity, distinct from whole colony bleaching or paling. Thus, colonies suffering spatially restricted paling/bleaching may not necessarily transition into full colony paling/bleaching and subsequent mortality, as indicated by the dashed lines. Likewise, spatially restricted and whole colony paling may not necessarily lead to bleaching. Quantifying bleaching in terms of the degree of algal symbiont loss (intensity) and spatial extent (bleached area) in conjunction with paling/bleaching dynamics over time will allow to resolve to what extent spatially restricted paling/bleaching results in full colony bleaching and in what cases it represents an ecologically distinct phenomenon. Created with BioRender.com under license number WM275BF87N.

A qualitative review of photographically documented reports of partial coral bleaching cases found 25 studies published between 1990 and 2024 (Table S1). This highlighted that the notion of “partial bleaching” is ambiguous. It can refer to colonies bleaching in a spatially restricted pattern (i.e., patchily amidst fully pigmented areas of the same colony, Figs. 1 and 2) or to colonies suffering homogeneous yet mild degrees of discoloration (i.e., paling) across their entire surface. Collectively, these studies document heat stress-induced partial bleaching, irrespective of the coral growth form, in 25 scleractinian coral species within 18 genera in different regions, including the Caribbean, Eastern Tropical Pacific, Western Pacific, Indian Ocean, and Central Indo-Pacific (Table S1). Partial bleaching patterns were not uniform across coral colonies or species (i.e., the position and size of the bleached and unbleached sections varied across conspecifics and reports). In the most extreme cases, bleaching affected neighboring polyps differentially (Fig. 1g). Importantly, none of these studies tracked the progression of partial bleaching over time to determine whether partially bleached corals recover or bleach completely in terms of spatial extent.

Studies tracking the progression of bleaching through single (tagged) coral colonies over time in situ are uncommon, yet they shed light on the order in which pigmentation is lost and could resolve whether some or all partially bleached coral colonies eventually bleach completely. Tracking the relative prevalence of partial vs. fully bleached colonies is, however, logistically demanding. It requires tagging and revisiting large numbers of colonies, given that the manifestation of one or the other response is unknown prior to the onset of bleaching. In the Abrolhos Archipelago (Brazil), for instance, several colonies of *Millepora alcicornis* bleached partially (30–90% of the colony) but became fully bleached or died with ongoing and accumulating heat stress^26^. The published studies documenting cases of partial bleaching reviewed here most likely represent a minuscule fraction of the cases that occur worldwide during the increasingly frequent mass bleaching events (Fig. 1). It is, however, clear that partial bleaching is occasionally reported from various species and sites, but is not contextualized as a phenomenon distinct from progressive whole-colony bleaching (whitening) over time, i.e., the time it takes for a colony to fully bleach. We consider coral bleaching where only certain parts of a colony bleach (or pale) as spatially restricted, distinct from whole-colony paling and bleaching (Fig. 2). In the following, we discuss the putative causes underlying spatially restricted bleaching and discuss methodologies to resolve whether these indeed constitute ecologically distinct phenomena.

## What drives differential bleaching susceptibility within a single coral colony?

When considering whether spatially restricted paling and bleaching (Figs. 1 and 2) is an ecological phenomenon distinct from whole-colony paling or discoloration (Fig. 1a), it is important to acknowledge that corals are colonial holobionts. Colonial refers to the fact that corals are composed of (at times) millions of identical building blocks, i.e., individual polyps, that can, to a certain extent, function independently from each other. Each polyp is a small, anemone-like organism, resembling an inverted jellyfish. A polyp consists of a mouth region surrounded by tentacles that capture food and trap particles and nutrients, a columnar body that comprises the gastrovascular cavity, and a basal region that attaches to the skeleton. Polyps within a colony may be physically connected by a tissue layer at the basal region known as the coenosarc. Thus, although polyps within a coral colony are independent units able to perform all essential biological functions, such as feeding and reproduction, they exchange signaling molecules and nutrients with one another through the coenosarc. Holobiont refers to the notion that each coral is composed of many organisms, including endosymbiotic algae of the family Symbiodiniaceae, associated bacteria, other eukaryotes, and viruses^27,28^. Different polyps or regions of a coral colony can be exposed to different extrinsic physical-chemical factors (e.g., light, temperature, flow, nutrient availability, oxygen concentrations)^29–31^, which we refer to in the following as “microenvironments”. By comparison, the term “microhabitats” refers to intrinsic (biological) differences across the colony surface (e.g., Symbiodiniaceae assemblage, bacterial microbiome, metabolites, etc.). The latter may be independent of, or consequential to, microenvironmental differences. In the following sections, we discuss the biological consequences of the different microenvironments that corals are exposed to and how they may contribute to the observed phenomenon of spatially restricted bleaching, which in the most extreme case can be at the scale of neighboring polyps (Fig. 1f) or even among tentacles of a single polyp.

### Heterogeneity in the Symbiodiniaceae assemblage

Endosymbiotic algae in the family Symbiodiniaceae produce sugars and other metabolites through photosynthesis, which provides the coral host with the energy to build their skeletons, which in turn provide habitat for many other species and give rise to the three-dimensional reefscape^32^. This intimate relationship is the engine of the reef^33,34^, and, unsurprisingly, the functional diversity among Symbiodiniaceae associated with a coral colony affects its stress tolerance and resilience^35,36^. The majority of coral species exhibit high partner fidelity and thus associate predictably with certain algal species^37–39^, with exceptions^40,41^. Based on this notion, it is typically assumed that a single algal genotype of a given symbiont species will dominate a single coral colony^42–44^. However, within-colony variability in Symbiodiniaceae distribution occurs in some corals and may explain why some areas of a colony bleach while others do not^31,45,46^. For instance, two studies on the dominant Caribbean corals *Orbicella annularis* and *Orbicella faveolata* found that these species can host multi-species Symbiodiniaceae communities. In the first study, this observation explained patterns of differential bleaching susceptibility^45^, while in the second study distinct zonation patterns of Symbiodiniaceae correlated with light availability, cardinal direction, and depth^46^. A third study collected a diverse range of 20 coral species (11 genera) in the Cook Islands, Australia, to assess whether distinct light environments across a colony surface permit Symbiodiniaceae cohabitation^31^. Based on ITS2 amplicon sequence variants (ASVs) or ITS2 type profiles, representative of distinct Symbiodiniaceae taxa^47^, the study could not unambiguously resolve different symbiont communities by light environments. In a fourth study, based on ITS2 type profiles, multiple putative C15 genotypes within *Porites* colonies were found, although they showed no clear structuring by environmental conditions^48^. Taken together, current studies support that harboring multiple genetically distinct and co-dominant species of Symbiodiniaceae is observed but rare, producing rather homogeneous symbiont communities among individuals of a given coral species.

Besides differences in algal association, Symbiodiniaceae density may vary across coral species^49^, across colonies of a given species^50^, and within coral colonies^45,46^. Higher densities of Symbiodiniaceae may protect the coral host from light stress^51^ and provide higher carbon translocation rates, thus increasing resilience^52^. However, experimental evidence is rather indirect. Two studies observed a decrease in Symbiodiniaceae density in the summer season. While the first study claims that an increased abundance of photosynthetic bacteria during summer may mitigate the putative adverse effects of reduced Symbiodiniaceae density^51^, the second study argues that increased photosynthetic activity compensates for the reduced symbiont cell density. Thus, although higher densities of Symbiodiniaceae could contribute to coral resilience by enhancing carbon translocation and providing some protection against light stress, the overall effect depends on a complex interplay of factors, including symbiont type, environmental conditions, and coral species. Conversely, more recent studies suggest that increased or stable Symbiodiniaceae densities under heat stress may be the first sign of an imbalanced nutrient cycling between the coral host and its symbiont algae, eventually leading to bleaching^6,53^. In the latter study^6^, the authors could show that concomitant with heat stress but before the onset of visual bleaching, the mitotic index of algal symbiont populations showed an approximately threefold increase, consistent with an enhanced nitrogen availability and increased retention of photosynthates, both of which are hallmarks of imbalanced nutrient cycling. Ceased carbon release by the algal symbiont is then suggested to be the trigger for its expulsion (or digestion) through inability to maintain an arrested phagosome state^6,54^.

Importantly, we are still naive with regard to our understanding of how fine-scale genetic differences in coral-algal symbiont pairings at the genotype level affect holobiont phenotypes. If corals are genetic mosaics (see below) and certain algal genotypes exhibit a higher degree of mutualism with one host genotype but not another, then the notion of clonal propagation of algal genotypes within colonies may affect parts of a colony (harboring polyps with different genotypes) differently, possibly contributing to spatially restricted bleaching. In light of this, spatially restricted bleaching may be the manifestation of inadequate symbiont functional diversity relative to the constraints presented by the coral and the microenvironment. In other words, the physical and/or chemical environment experienced by Symbiodiniaceae cells (e.g., light, nutrients, oxygen) may differ widely across the surface of the colony, which in turn will impact algal physiology^55^.

### Heterogeneity in coral holobiont-associated bacteria and archaea

The three-dimensional structure of a coral colony as well as the different compartments of the coral host (e.g., mucus, tissue, skeleton) create a myriad of distinct microenvironments and microhabitats that structure the holobiont microbiome^28^. The bacterial assemblage across the surface of a single coral colony can be highly heterogeneous and distinct across the different coral compartments^56–58^. These bacterial communities support the existence of corals in oligotrophic waters by contributing to nutrient cycling, dissolved organic matter (DOM) processing, protection against pathogens, and stress resilience^59–62^. Bacteria are thus integral components of the coral holobiont, contributing to its functioning, resilience, and adaptability^63^. Therefore, it is reasonable to assume that compositionally distinct microbiome assemblages across the colony surface give rise to functional differences, with consequences for how certain areas of a colony may react to thermal stress, i.e., bleach. In this regard, a recent study on coral-associated microbial aggregates (CAMAs) within tissues of the coral *Acropora hyacinthus* could show that CAMAs were differentially distributed across the colony and tissue types and hosted different bacterial morphotypes, suggesting spatial and functional specialization across the coral colony^64^. Less well understood, however, are the dynamics of bacterial communities in relation to their spatial position in the coral holobiont. For instance, one may hypothesize that the top of a colony is subject to more intense and variable UV exposure than the sides of a colony, with putative consequences for bacterial community dynamics across days, months, and seasons^65^. In addition to such considerations, the bacterial community composition is likely influenced by the presence of specific Symbiodiniaceae, with several studies showing that different algal symbionts harbor specific microbiomes *in hospite* and in culture, based on amplicon and metagenomic sequencing as well as fluorescent microscopy^66–70^. Hence, heterogeneity in the Symbiodiniaceae assemblage is another structuring factor of bacterial communities, and those coral species with more flexible algal associations should exhibit more plastic bacterial communities, the consequences of which are yet to be resolved.

### ***Heterogeneity in the assemblage of viruses, fungi, endolithic algae, and microeukaryotes***

As for bacteria and archaea, the distribution of viruses, fungi, endolithic algae, and microeukaryotes may co-vary across a coral colony. With many coral-associated viruses being prophages (i.e., bacteria-associated), it follows that spatially varying bacterial assemblages result in varying viral assemblages^28,71,72^. In addition, Symbiodiniaceae-associated viruses are directly affected by heat stress and may contribute to their thermal sensitivity, which is consequential to coral bleaching susceptibility^73,74^. Fungal diversity can vary across different parts of a single coral colony, and spatial differences in fungal communities have been noted within coral tissues and the surrounding mucus layer^75,76^. Endolithic algae are microorganisms that live inside the porous skeletons of corals, are primarily photosynthetic, and include cyanobacteria, green algae (Chlorophyta), and occasionally diatoms, most notably *Ostreobium*^77–80^. The primary factor affecting their distribution is light availability. These algae are thus typically found in the upper layers of the coral skeleton where light penetration is sufficient for photosynthesis. Deeper regions of the skeleton and consequently deeper (or shaded) parts of a coral colony receive less light and thus harbor fewer photosynthetic endoliths^77,79^. During coral bleaching, endolithic algae may provide an alternative source of energy for the coral^77,79,81^. Experimental support comes from measuring increased endolith density and carbon translocation dynamics in bleached and non-bleached corals of *Oculina patagonica*^81^. Conversely, a more recent study found that bleaching-resistant *Goniastrea edwardsi* corals were associated with endolithic microbiomes of greater functional diversity and redundancy that exhibited lower nitrogen and carbon assimilation than endoliths in bleaching-sensitive *Porites lutea* colonies^78^. This is potentially due to the stabilizing effect on retaining nutrient-limiting conditions characteristic of stable coral-algal symbioses, although carbon translocation from the skeleton to the tissue was not measured. Thus, besides contributing to bleaching survival by providing an alternative source of energy, endolithic microbiomes may also stabilize nutrient fluxes in the coral holobiont to maintain homeostasis under thermal stress.

### Heterogeneity across the molecular phenotypes of coral polyps

We refer to the “molecular phenotype” within coral polyps as the umbrella term that encompasses their “elementome”, “metabolome”, and “(small) signaling molecules”^82^. These entities are highly dynamic and reflective of the processes at play at the time of coral sampling or observation. The elementome, defined here as the quantity and proportion of elements a coral or polyp requires to grow and survive^83^, determines its biogeochemical niche^84,85^. These bioelements include macroelements such as carbon, nitrogen, phosphorus, sulfur, calcium, and magnesium, as well as trace elements like strontium, iron, molybdenum, manganese, and zinc. Besides their presence, the ratios in which they occur (i.e., their stoichiometry) are crucial to maintaining the structural integrity of biomolecules, metabolic efficiency, and homeostasis^85,86^.

Closely linked to coral elementomes, coral metabolomes comprise the chemical array of end-products of cellular processes (e.g., amino acids, sugars, lipids, nucleotides, and secondary metabolites), the profiling of which provides a snapshot of the metabolic physiological state of corals^87–89^. Importantly, the bleaching history of a coral leaves a strong metabolomic signature in both the host and Symbiodiniaceae, possibly even a few years after exposure to thermal stress^90^. Small signaling molecules play key roles in intra- and extra-cellular communication by activating pathways and mediating external signals. For instance, signaling molecules such as glycoproteins, lipids, and peptides regulate the coral-algal symbiosis^91^, reactive oxygen species trigger the activation of heat stress responses and cellular repair pathways^5,92^, and the proteobacterial compound tetrabromopyrrole (TBP) induces coral larval settlement and metamorphosis^93^. Given the presence of different microenvironments within coral colonies^94^, partially dictated by the nature of the colonial setup with polyps as the basic building unit, we expect molecular phenotypes to vary across coral surfaces and posit that this variation may underpin intra-colonial differences in vulnerability and response to thermal stress^94^. Nutrient requirements in terms of nutrient species and quota differ within coral colonies, which means that the polyps of a colony and their Symbiodiniaceae live in different nutrient statuses^95^. These intra-colony differences in elementomes are further exacerbated as thermal stress destabilizes symbiotic nutrient cycling in corals^6,53^, possibly contributing to spatially restricted bleaching within coral colonies. The extent to which elementomes vary spatially within and across coral colonies, potentially governing their responses to thermal stress, remains, however, largely untested^96^.

### Somatic mutations, epigenetic control, and chimerism contribute to a heterologous coral genome across the colony surface

The importance of somatic mutations, especially for animals with long generation times (e.g., corals), has been previously discussed and proposed as an overlooked component in coral adaptation^25^. This importance is attributed to at least four observations: (i) somatic mutation rates may be orders of magnitude higher than germ line mutation rates^97,98^, (ii) corals can reproduce asexually through fragmentation, which may give rise to colonies with altered genotypes due to intracolonial genetic variability generated by somatic mutations^25,99–103^ (although it is unknown how common such phenomena are), (iii) endosymbiotic Symbiodiniaceae, which are haploid in the vegetative state, are subject themselves to somatic mutations and reproduce through cell division, and (iv) somatic mutations can enter the germline (it is unclear whether corals harbor distinct germ cells) and are inherited by coral larvae^99^. Likewise, gene-body methylation (gbM) as a mechanism of epigenetic control^104^ can change in response to ecological stress and has been shown to vary across the coral colony surface^105^.

In the context of spatially restricted bleaching, somatic mutations (of the coral host and algal symbionts) and differential gbM cause genetic mosaicism that may fuel intra-colony phenotypic variation and produce heterogeneity in colony surface bleaching^25^, with previous estimates arguing for somatic mutations and gbM differences being abundantly present in colonies^25,99,105^. Distinct from genetic mosaicism is coral chimerism, where closely related individuals fuse, resulting in multiple distinct genotypes within a colony. This fusion is readily observed following the gregarious settlement of coral recruits in aquaculture settings^106^, but potential chimeras have also been detected in adult colonies in the wild^107^. It is thus expected that, even in the absence of clear microenvironmental differences or gradients across the colony surface, coral chimerism is likely to result in pronounced patterns of spatially restricted bleaching.

## What are the consequences of differential bleaching susceptibility within a single coral colony?

**Fig. 3:**
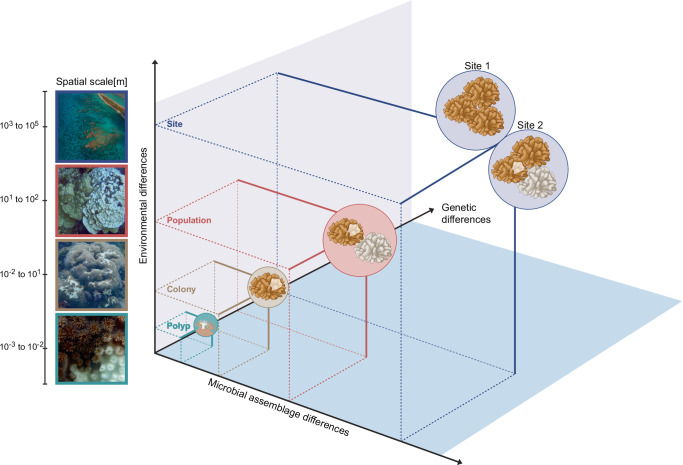
Scales of environmental variability and holobiont complexity underlying heterogeneity in the response to thermal stress, putatively leading to spatially restricted bleaching. The illustration depicts the different scales [m] of coral holobionts, from polyps to colonies to populations to sites. Heterogeneity in bleaching susceptibility may occur at the scale of coral polyps (10^−^^3^ to 10^−^^2 ^m, i.e., from mm to cm), across neighboring sections within a coral colony (10^−^^2^ to 10^1 ^m, i.e. from cm to m), across neighboring colonies of the same species (i.e., 10^1^ to 10^2^m, i.e., across meters), and across reef sites (10^3^ to 10^5 ^m, i.e., across km). Thus, scales of environmental variability and holobiont complexity span eight orders of magnitude. Across scales, environmental (e.g., light, temperature, flow, nutrient availability, and oxygen concentrations), genetic (e.g., somatic mutation, gbM), and microbial assemblage (e.g., Symbiodiniaceae, bacteria, archaea, viruses, fungi, endolithic algae, and microeukaryotes) differences are assumed to increase in magnitude and all contribute to differences in the thermal stress response, i.e., bleaching susceptibility. Images by (from top to bottom): Tara Pacific, Sebastian Szereday, Emma Camp, and Sonia Bejarano. Draft figure created with BioRender.com under license number SD275BFO9Z.

Having discussed putative causes underlying spatially restricted bleaching, the ecological consequences of spatially restricted bleaching are unknown (Fig. 3). One may argue that bleaching-resistant areas constitute coral colony refugia. It is, however, unknown to what extent this would affect colony growth patterns and morphology. It is also unclear whether spatially restricted bleaching is associated with higher or lower bleaching-associated mortality or recovery, besides the population or reef-level consequences. A previous study following the bleaching of various coral species found that whole-colony mortality was generally higher in Acroporids than in *Platygyra daedalea* and *Porites lobata*^108^. The latter two suffered mostly partial mortality (i.e., spatially restricted tissue loss) but few colonies died^108^. This might indicate that partial bleaching may be a mechanism for colony survival, with colony size and/or growth form affecting the propensity for spatially restricted bleaching.

Disentangling whether spatially restricted bleaching is a larger phenomenon of intra-colony heterogeneity in bleaching susceptibility or simply part of progressive “classic” bleaching (i.e., transitory) (Fig. 2) requires repeated observations spanning recurring bleaching events as well as multiple sampling locations within a colony. Such observations are ideally guided by in situ temperature measurements, with coral species exhibiting planar growth forms potentially being good model systems, as they are presumably exposed to more similar microenvironments. Time-resolved bleaching studies that ideally track the onset and spatial dynamics of paling/bleaching across coral colonies in a reef environment are necessary to provide answers to such questions. In this context, 3D photogrammetry addresses some of the difficulties in tracking single coral colonies in cases when partial mortality causes fission and fusion over time^109,110^ and, coupled with machine learning-based tools (e.g., TagLab), has proven useful^111^. Accompanying such imaging approach by non-intrusive microsampling e.g.^112^ could shed light on whether intra-colony variability in bleaching susceptibility aligns with microhabitat differences in the holobiont between bleached and unbleached parts of a colony.

Alternatively, acute heat stress assays that provide standardized phenotype diagnostics (e.g., the Coral Bleaching Automated Stress System, CBASS)^113,114^ probing different regions across the colony surface could be employed to test whether spatial differences in thermal tolerance exist. In conjunction with the sequencing of probed fragments, thermal tolerance differences could be aligned to the algal and microbiome assemblages or differences in the epigenetic/genetic composition of the coral host and algal symbionts. CBASS assays could, for instance, be run on ramets spread across, or clustered within, a colony. Such assays could follow hypotheses regarding putative differences between areas exposed to different microenvironments within a colony (e.g., lit and shaded areas) and be applied to colonies exhibiting various growth forms. Further, once bleached and unbleached regions across the colony surface emerge during a bleaching event, prior acute heat stress assay testing could reveal the extent to which higher and lower ED50 values (i.e., standardized thermal tolerance thresholds) align with decreased or increased bleaching susceptibility. A previous study found that northern Red Sea corals with lower ED50s showed a strong, temperature-induced gene expression response, indicating resilience^15^. In contrast, central Red Sea corals with higher ED50s had consistently elevated gene expression levels, suggesting a thermally resistant population that cannot further attune. Such ability to mount a rapid and pervasive gene expression response conditioned to the amplitude and duration of thermal stress was termed transcriptomic resilience and acclimation^115^. Further, since it is currently unclear what ecological phenomenon is best reflected by ED50 values, CBASS assay testing could accompany recovery surveys to track whether recovery ability or rates align with higher or lower ED50s^116^.

In general, the alignment of in situ thermal stress data with CBASS experimental stress temperatures could help to better understand the relationship between experimental responses and a coral’s natural bleaching susceptibility and capacity to recover. Further to CBASS testing, if coupled with an MTP system or imaging PAM finer-scale intra-nubbin/fragment differences could also be revealed and tracked^117^. Additionally, microsensors are well-suited to resolve microenvironmental differences and the consequential intracellular differences, such as reactive oxygen species levels^55,118^, in addition to elemental mapping across the colony surface and algal symbionts^68,94^.

### Integrating a colony and polyp perspective into coral research

In the sections above, we have touched upon research directions and approaches that would unequivocally establish the presence and prevalence of coral surface heterogeneity and its alignment with intra-colony differences in thermal tolerance and bleaching. Here we specifically discuss the consequences implicit in the premise that coral colonies exhibit spatially differential susceptibility to bleaching. First, it challenges the common assumption underlying experimental biology and restoration efforts that coral fragments are representative of the sampled coral colony at large^14^. We have limited knowledge of the potential biases introduced by working with fragments of colonies. If spatial differences across the coral colony surface exist, then it matters which part of the colony was used for a heat stress experiment or as a fragment for restoration purposes. Notably, these differences are not solely due to acclimation, as different parts of a colony may harbor different coral and algal genotypes. Whether newly-formed polyps should be treated as separate entities remains unclear; further research is required to determine whether the local microenvironment at the time of budding may affect symbiotic assemblages or nutritional status. At large, it will be critical to understand the relative contribution of adaptive and acclimatory differences across the coral colony surface. Thus, it will be valuable to understand the extent to which genetic variability occurs and correlates with differences in thermal tolerance and bleaching susceptibility across the coral colony surface. In addition, it will be important to understand how microbial community composition and abundance vary spatially across the surface of a colony, how this variability affects thermal tolerance, and to what extent it is subject to acclimation. Answers to these questions hold important considerations for experimental biology (i.e., the need for within-colony replicates) and restoration (i.e., the importance of spatially explicit sampling of targeted colonies to preserve targeted traits, such as superior thermal tolerance).

The complexity of the coral holobiont and its impact on coral thermal tolerance are acknowledged^5,15,119,120^. Adopting a single-colony perspective can clarify how differences among colonies arise and what factors underlie population variance, as the latter is the raw material for natural selection and restoration^12,121,122^. Similarly, the single-polyp scale provides a within-colony colony perspective to acknowledge the independence (and interdependence) of the building units of coral colonies, which may be understood as holobionts of an emergent colony meta-holobiont. Integrating bleaching research across the scales of polyp, colony, population, and species will increase insight towards a conceptual framework that will advance coral reef science and active intervention^10,27^.

### Reporting summary

Further information on research design is available in the Nature Portfolio Reporting Summary linked to this article.

## Supplementary information

Supplementary Information

Reporting Summary
