# Supplementary material for: Spatially restricted coral bleaching as an ecological manifestation of within-colony heterogeneity
Source: Commun Biol. 2025 May 13;8:740. doi: 10.1038/s42003-025-08150-4 (PMC12075583; doi:10.1038/s42003-025-08150-4)
Supplement: Supplementary file 1 — Supplementary Information [file 42003_2025_8150_MOESM1_ESM.pdf]

Spatially restricted coral bleaching as an ecological manifestation of within-colony heterogeneity

Christian R. Voolstra[1,+,*], Marlen Schlotheuber[1,+], Emma F. Camp[2], Matthew R. Nitschke[3,4], Sebastian Szereday[5], Sonia Bejarano[6,+,*]

[1] Department of Biology, University of Konstanz, Konstanz, Germany.
[2] Climate Change Cluster, University of Technology Sydney, Broadway, New South Wales, Australia.
[3] Australian Institute of Marine Science, Townsville, Australia
[4] School of Biological Sciences, Victoria University of Wellington, Wellington, New Zealand
[5] Coralku Solutions, Non-Profit Organization for Coral Reef Research and Restoration, Kuala Lumpur, Malaysia
[6] Reef Systems Research Group, Leibniz Centre for Tropical Marine Research (ZMT), Bremen, Germany.

[+] contributed equally, *corresponding authors

Content

Table S1. References retrieved through a qualitative review of photographically documented reports of partial coral bleaching cases.

References

Table S1. References retrieved through a qualitative review of photographically-documented reports of partial coral bleaching cases, including references from 1990 to March 2024.

Reference	Species suffering partial bleaching	Region
Goreau and McFarlane (1990)	*Orbicella annularis*	Jamaica (Caribbean)
Goreau and Hayes (1994)	*Montastraea cavernosa*	Jamaica (Caribbean)
Glynn (1996)	*Colpophyllia natans*	Puerto Rico (Caribbean)
	Agaricia agaricites	Belize (Caribbean)
	Goniastrea retiformis	Japan (Pacific)
Rowan et al (1997)	*Orbicella annularis*	Panama (Caribbean)
	Orbicella faveolata	Panama (Caribbean)
Edmunds et al (2003)	*Orbicella franksi*	Florida keys (Caribbean)
McClannahan (2004)	*Porites lutea*	Kenya (Indian Ocean)
Sotka and Thaker (2005)	*Montipora* spp.	Australian Great Barrier Reef (Central Indo-Pacific)
Baker et al (2008)	*Orbicella faveolata*	Panama (Caribbean)
	Porites lobata	Easter Island (Pacific Ocean)
	Pocillopora spp.	Easter Island (Pacific Ocean)
	Pavona clavus	Panama (Eastern Tropical Pacific)
Brandt et al (2009)	*Colpophyllia natans*	Florida keys (Caribbean)
	Siderastrea siderea	Florida keys (Caribbean)
van Oppen et al (2011)	*Montipora digitata*	Not specified
	Goniopora sp.	Not specified
	Lobophyllia hemprichii	Not specified
Kemp et al (2014)	*Orbicella faveolata*	Mexico (Caribbean)
Kemp et al (2015)	*Orbicella faveolata*	Mexico (Caribbean)
Pinzon et al (2015)	*Orbicella faveolata*	Puerto Rico (Caribbean)
Chow et al (2016)	*Porites* sp.	Hong Kong (Pacific Ocean)
Neal et al (2017)	*Orbicella franksi*	Panama (Caribbean)
	Stephanocoenia michellini	Panama (Caribbean)
	Siderastrea siderea	Panama (Caribbean)
Patterson et al (2018)	*Porites* sp.	Gulf of Mannar (Indian Ocean)
Durante et al (2019)	*Acropora palmata*	Florida keys (Caribbean)
McClannahan et al (2020)	*Montipora* sp.	Not specified
Ramesh et al (2020)	*Porites lutea*	India (Indian Ocean)
Ip et al (2022)	*Montipora peltiformis*	Hong Kong (Pacific Ocean)
	Pavona decussata	Hong Kong (Pacific Ocean)

Table S1 (continued). References retrieved through a qualitative review of photographically-documented reports of partial coral bleaching cases, including references from 1990 to March 2024.

**Reference**	**Species suffering partial bleaching**	**Region**
De et al (2022)	*Porites lichen*	India (Arabian Sea)
	Porites compressa	India (Arabian Sea)
	Favites melicerum	India (Arabian Sea)
	Turbinaria mesenterina	India (Arabian Sea)
	Pseudosiderastrea tayami	India (Arabian Sea)
	Cyphastrea serailia	India (Arabian Sea)
	Plesiastrea versipora	India (Arabian Sea)
	Goniopora sp.	India (Arabian Sea)
	Siderastrea savignyana	India (Arabian Sea)
Lopez-Londoño et al (2024)	*Orbicella faveolata*	Mexico (Caribbean)
Linsmayer et al (2024)	*Orbicella franksi*	Panama (Caribbean)
Zou et al (2024)	*Montipora peltiformis*	Hong Kong (Pacific Ocean)
	Pavona decussata	Hong Kong (Pacific Ocean)
Zhang et al (2024)	*Pavona decussata*	South China Sea (Pacific Ocean)

References

Baker, A. C., Glynn, P. W., & Riegl, B. (2008). Climate change and coral reef bleaching: An ecological assessment of long-term impacts, recovery trends and future outlook. *Estuarine, Coastal and Shelf Science, 80*(4), 435–471.

Brandt, M. E. (2009). The effect of species and colony size on the bleaching response of reef-building corals in the Florida Keys during the 2005 mass bleaching event. *Coral Reefs, 28*(4), 911–924.

Chow, M. H., Tsang, R. H. L., Lam, E. K. Y., & Ang, P. (2016). Quantifying the degree of coral bleaching using digital photographic technique. *Journal of Experimental Marine Biology and Ecology, 479,* 60–68.

De, K., Nanajkar, M., Arora, M., Nithyanandan, M., Mote, S., & Ingole, B. (2022). Application of remotely sensed sea surface temperature for assessment of recurrent coral bleaching (2014–2019) impact on a marginal coral ecosystem. *Geocarto International, 37*(15), 4483–4508.

Durante, M. K., Baums, I. B., Williams, D. E., Vohsen, S., & Kemp, D. W. (2019). What drives phenotypic divergence among coral clonemates of *Acropora palmata*? *Molecular Ecology, 28*(13), 3208–3224.

Edmunds, P. J., Gates, R. D., & Gleason, D. F. (2003). The tissue composition of Montastraea franksi during a natural bleaching event in the Florida Keys. *Coral Reefs, 22*(1), 54–62.

Edward, J. K. P., Mathews, G., Raj, K. D., Laju, R. L., Bharath, M. S., Arasamuthu, A., Kumar, P. D., Bilgi, D. S., & Malleshappa, H. (2018). Coral mortality in the Gulf of Mannar, southeastern India, due to bleaching caused by elevated sea temperature in 2016. *Current Science, 114*(9), 1967–1972.

Glynn, P. W. (1996). Coral reef bleaching: facts, hypotheses and implications. *Global Change Biology, 2*(6), 495–509.

Goreau, T. J., & Hayes, R. L. (1994). Coral bleaching and ocean "hot spots". *Ambio, 23*(3), 176–180.

Goreau, T. J., & Macfarlane, A. H. (1990). Reduced growth rate of *Montastrea annularis* following the 1987–1988 coral-bleaching event. *Coral Reefs, 8*(4), 211–215.

Ip, J. C.-H., Zhang, Y., Xie, J. Y., Yeung, Y. H., & Qiu, J.-W. (2022). Stable Symbiodiniaceae composition in three coral species during the 2017 natural bleaching event in subtropical Hong Kong. *Marine Pollution Bulletin, 184*, 114224.

Kemp, D. W., Hernandez-Pech, X., Iglesias-Prieto, R., Fitt, W. K., & Schmidt, G. W. (2014). Community dynamics and physiology of *Symbiodinium* spp. before, during, and after a coral bleaching event. *Limnology and Oceanography, 59*(3), 788–797.

Kemp, D. W., Thornhill, D. J., Rotjan, R. D., Iglesias-Prieto, R., Fitt, W. K., & Schmidt, G. W. (2015). Spatially distinct and regionally endemic Symbiodinium assemblages in the threatened Caribbean reef-building coral *Orbicella faveolata*. *Coral Reefs, 34*(2), 535–547.

Linsmayer, L. B., Noel, S. K., Leray, M., Wangpraseurt, D., Hassibi, C., Kline, D. I., & Tresguerres, M. (2024). Effects of bleaching on oxygen dynamics and energy metabolism of two Caribbean coral species. *Science of the Total Environment, 919*, 170753.

López-Londoño, T., Enríquez, S., & Iglesias-Prieto, R. (2024). Effects of surface geometry on light exposure, photoacclimation and photosynthetic energy acquisition in zooxanthellate corals. *PloS One, 19*(1), e0295283.

McClanahan, T. R. (2004). The relationship between bleaching and mortality of common corals. *Marine Biology, 144*(6), 1239–1245.

McClanahan, T. R., Darling, E. S., Maina, J. M., Muthiga, N. A., D'agata, S., Leblond, J., Arthur, R., Jupiter, S. D., Wilson, S. K., Mangubhai, S., Ussi, A. M., Guillaume, M. M. M., Humphries, A. T., Patankar, V., Shedrawi, G., Pagu, J., & Grimsditch, G. (2020). Highly variable taxa-specific coral bleaching responses to thermal stresses. *Marine Ecology Progress Series, 648*, 135–151.

Neal, B. P., Khen, A., Treibitz, T., Beijbom, O., O'Connor, G., Coffroth, M. A., Knowlton, N., Kriegman, D., Mitchell, B. G., & Kline, D. I. (2017). Caribbean massive corals not recovering from repeated thermal stress events during 2005-2013. *Ecology and Evolution, 7*(5), 1339–1353.

Patterson Edward, J. K.; Mathews, G.; Diraviya Raj, K.; Laju, R. L.; Selva Bharath, M.; Arasamuthu, A.; Dinesh Kumar, P.; Bilgi, D. S.; Malleshappa, H. (2018) Coral mortality in the Gulf of Mannar, Southeastern India, due to bleaching caused by elevated sea temperature in 2016. *Current Science, 114* (09), 1967.

Pinzón, J. H., Kamel, B., Burge, C. A., Harvell, C. D., Medina, M., Weil, E., & Mydlarz, L. D. (2015). Whole transcriptome analysis reveals changes in expression of immune-related genes during and after bleaching in a reef-building coral. *Royal Society Open Science, 2*(4), 140214.

Ramesh, C.; Koushik, S.; Shunmugaraj, T.; Murthy, M. V. R. (2020) Coral colors as a heat stress indicator during bleaching events. *Journal of Wildlife Research*, *8*(3), 68-70.

Rowan, R., Knowlton, N., Baker, A., & Jara, J. (1997). Landscape ecology of algal symbionts creates variation in episodes of coral bleaching. *Nature, 388*(6639), 265–269.

Sotka, E. E., & Thacker, R. W. (2005). Do some corals like it hot? *Trends in Ecology & Evolution, 20*(2), 59–62.

Van Oppen, M. J. H., Souter, P., Howells, E. J., Heyward, A., & Berkelmans, R. (2011). Novel Genetic Diversity Through Somatic Mutations: Fuel for Adaptation of Reef Corals? *Diversity, 3*(3), 405–423.

Zhang, M., Huang, S., Luo, L., Yu, X., Wang, H., Yu, K., & Zhong, S. (2024). Insights into the molecular mechanisms underlying the different heat tolerance of the scleractinian coral *Pavona decussata*. *Coral Reefs, 43*(2), 429–442.

Zou, Y., Ip, J. C.-H., Xie, J. Y., Yeung, Y. H., Wei, L., Guo, Z., Zhang, Y., & Qiu, J.-W. (2024). Dynamic changes in bacterial communities in three species of corals during the 2017 bleaching event in subtropical Hong Kong waters. *Marine Pollution Bulletin, 199,* 116002.
